# Carbon Impurity Entrapping and Charge Localization within TiO_2_ Nanoparticle Films

**DOI:** 10.1021/acs.jpcc.5c04882

**Published:** 2025-10-29

**Authors:** Guillem Vives Ollé, Gilles R. Bourret, Thomas Berger, Oliver Diwald

**Affiliations:** Department of Chemistry and Physics of Materials, 625366 Paris-Lodron University Salzburg, Jakob-Haringer-Straße 2a, A-5020 Salzburg, Austria

## Abstract

Titanium dioxide (TiO_2_) particle systems are well-established photocatalysts with high performance under UV irradiation. They are often used as supported nanostructured thin films composed of interconnected TiO_2_ nanoparticles. During the film preparation, a variety of defects can be introduced, which can have a significant influence on the material performance. This can be used for defect engineering to enhance charge generation and separation within photocatalysts. We report here a study of the paramagnetic properties of four different TiO_2_ nanoparticle architectures. The spin concentrations measured on supported films and free-standing nanoparticles, in the presence or absence of dense TiO_2_ thin films prepared via sputtering, are compared. Organic additives are typically used for the immobilization of powdered photocatalyst materials or the production of photoelectrodes. Despite extensive cleaning and oxidative treatment for all cases where nanoparticle aggregation can occur or interfaces can form between the particles and the silicon substrate, paramagnetic carbon-related defects appear and become part of the lattice. In the concentration range of a few parts per million, underlying *carbonecks* act as electron traps and represent a previously overlooked defect type that may determine the photoelectronic properties of TiO_2_-based nanostructures.

## Graphical abstract

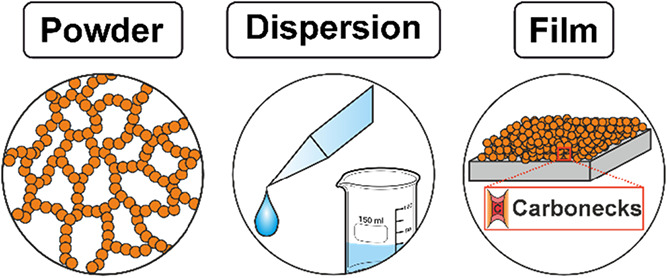

## Introduction

Nanostructured metal oxide films have attracted lots of attention for applications in sensing, solar fuel generation, water decontamination, and energy storage.^1–4^ They are often prepared by coating a substrate with a metal oxide nanoparticle slurry, which, after annealing, leads to an interconnected network of oxide nanoparticles.^5^ Because of its excellent and well-studied photocatalytic properties, such as high chemical stability, low cost, and nontoxic nature, titanium dioxide (TiO_2_) is the widely used material to prepare such films.^6^ The solar-to-charge carrier conversion efficiency of mesoporous TiO_2_ films can be strongly influenced by defects that form during the nanoparticle synthesis and the film preparation.^7,8^ Thus, knowing at what stage of the material formulation these defects form is key to enhancing the photocatalytic and photoelectrocatalytic properties of nanostructured oxide thin films.

Investigating the nature, concentration, influence, and fate of defects within metal oxide nanoparticles can be done using electron paramagnetic resonance (EPR) spectroscopy under both dark conditions and illumination. Indeed, light-induced charge separation processes generate paramagnetic states in solids that correspond to localized charge carriers and can be identified, analyzed, and interpreted using EPR.^1,9–13^

A recent study has shown that a particular type of extrinsic point defect associated with the presence of carbon impurities inside the TiO_2_ lattice, which we name *carboneck* center, forms predominantly in aggregated TiO_2_ nanoparticle systems and traps electrons.^10,14^ The associated paramagnetic center has a *g*-factor between *g* = 2.0018 and 2.0028, i.e., exactly in the magnetic field region where so-called color centers resonate in TiO_2_. A critical analysis of the combined structural characterization and EPR data suggests that the paramagnetic defect accumulates in the TiO_2_ nanoparticle necks and solid–solid interfaces during material processing.^10^ Density functional theory (DFT) calculations show that residual carbon atoms, possibly originating from synthesis, can substitute oxygen ions in the anionic sublattice, where they trap one or two electrons that are localized mainly at the carbon.^10^

The occurrence of substitutional carbon impurities during the formation of *carboneck* centers can be explained by the fact that all dynamic processes that occur during particle ensemble purification and oxidative carbon removal at elevated temperatures also involve the attachment and fusion of the particles. As a result, traces of carbon are trapped in the contact area between the particles, which transform from surface into bulk species upon particle coalescence. Even after extensive chemical and thermal post-treatment steps, carbon can remain in TiO_2_ nanomaterials at a concentration level of a few ppm.^15^ In the case of gas-phase-grown crystalline TiO_2_ nanoparticles with size distributions between 10 and 20 nm, which were subjected to extensive post-treatment in alternating gas atmospheres (O_2_ or vacuum), residual concentrations of carbon atoms in the range between 1 and 10 ppm were indirectly determined with EPR spectroscopy.

Spectroscopic characterization studies of defects in nanoparticle powders benefit from the usually high concentration of reactive sites and spectroscopically observable centers. Thin and smooth films, which are made from such nanoparticles and deposited on defined substrates such as single-crystal silicon, typically require a great deal of chemical formulation work. This includes additives, their later elimination, and final calcination of the entire structure. Related processing steps aim at thin nanoparticle layers that contain significantly less identifiable sites to be measured with an acceptable S/N ratio. Moreover, each processing step may in principle lead to the decrease or increase of defect concentrations.

We study here nanostructured TiO_2_ nanoparticle films prepared via conventional drop casting on Si substrates and dense TiO_2_ thin films. Defect characterization on thin nanostructured oxide films using EPR spectroscopy are highly relevant for photoelectrode design and photoactive surface layers. Such studies, however, are experimentally demanding because of the low number of active sites present in thin films compared to nanoparticle powder samples.

To the best of our knowledge, the underlying transfer of knowledge from powders to thin metal oxide nanoparticle films is still missing from the literature. Our EPR results including the quantitative assessment of spins show that even the most stringent material processing conditions cannot eliminate carbon impurities completely. They form extrinsic point defects that can incorporate into the lattice and emerge as charge traps and *carboneck* centers.

## Methods

### Sample Preparation

TiO_2_ nanoparticles are synthesized via metal–organic chemical vapor synthesis (MOCVS) (Figure 1a) in a flow reactor, as described by Siedl et al. Titanium isopropoxide (Sigma-Aldrich, 97%) is placed into the preheating zone at 383 K while the furnace temperature is kept at 1073 K.^3^ To maintain an inert atmosphere, argon is used as a carrier gas with a flow rate of 840 sccm. The overall pressure is kept at 30 mbar during the synthesis. The as-synthesized nanoparticle powder is annealed in oxygen and vacuum to remove carbon remnants and residual water from the particle surfaces. Heating rates, dwell times, and environmental gas atmospheres are as follows: first, the powder is annealed under high vacuum conditions to 873 K using a heating rate of 10 K·min^–1^. This temperature is held for 60 min under continuous pumping *p* < 10^–5^ mbar. Finally, 650 mbar of oxygen is introduced for 30 min, followed by evacuation for an additional 30 min. The oxygen admission–evacuation cycle is repeated two times. After a final oxygen admission step, the sample is cooled down in an oxygen atmosphere to *T* < 493 K in order to achieve a stoichiometric composition inside the TiO_2_ nanoparticle powder and stored under high vacuum conditions (*p* < 10^–5^ mbar) at room temperature.

**Figure 1:**
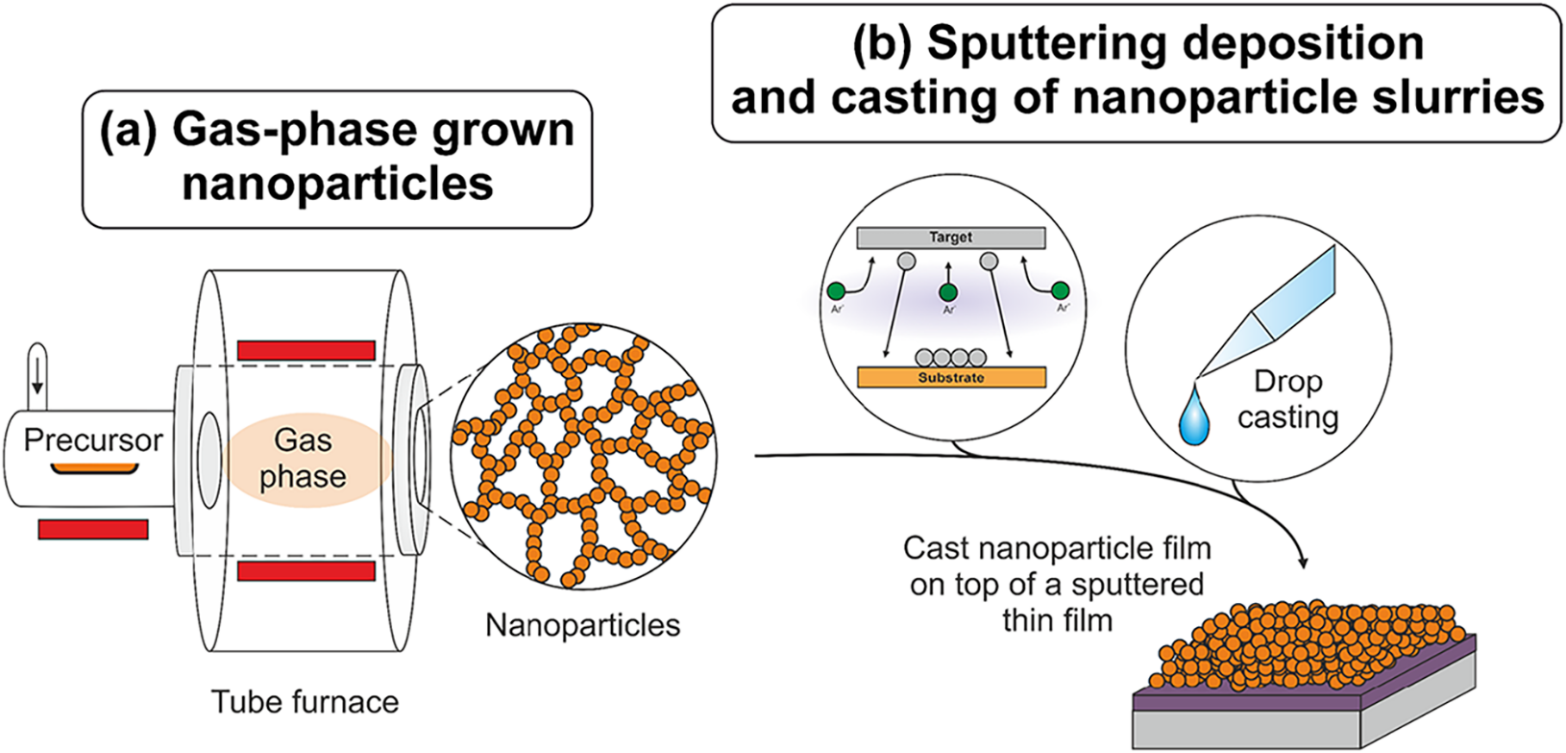
Schematic overview of the synthesis of (a) TiO_2_ nanoparticles and (b) a composite structure formed by a cast TiO_2_ nanoparticle film on top of a sputtered TiO_2_ film.

TiO_2_ nanoparticle films are prepared via drop casting. For this, 120 μL of Triton-X (Sigma-Aldrich, laboratory grade) is dissolved in 3.2 mL of distilled water. Subsequently, 120 μL of acetylacetone (Sigma-Aldrich, 99%) is added to the solution. Finally, 2.5 mg of annealed TiO_2_ nanoparticles are dispersed in 100 μL of the aqueous solution using a mortar to obtain a well-homogenized dispersion. The nanoparticle film is prepared by drop casting 10 μL·cm^–2^ of the nanoparticle dispersion onto the silicon substrate. After drying at room temperature, the film is placed in a muffle furnace at 723 K for 1 h, with an oxygen flow of 90 ft^3^·h^–1^. The heating rate is 5 K·min^–1^.

To obtain a composite structure (Figure 1b) consisting of a TiO_2_ nanoparticle film on top of a dense TiO_2_ film, silicon substrates were covered with a sputtered TiO_2_ layer prior to drop casting of the nanoparticle dispersion.

Dense TiO_2_ thin films are prepared using reactive sputtering in a Clustex100 M sputtering system from Leybold, Inc. on 3 × 0.3 cm^2^ single-crystalline, undoped, intrinsic silicon (Si-Mat, intrinsic, ⌀ 50.8 mm, <100>, resistance: ρ > 10^4^ Ohm·cm) pieces. The as-purchased silicon wafer reveals only a very weak EPR signal with *g*_iso_ = 2.0052 due to paramagnetic defects located at the SiO_2_/Si interface (Figure S1a).^16,17^ A SiO_2_ layer forms naturally due to the exposure of the silicon wafer to ambient conditions. Importantly, these defects are healed through vacuum annealing at 973 K (Figure S1b). Upon reoxidation of the silicon surface in an oxygen atmosphere at 873 K, this signal reappears only to a very minor extent (Figure S1c). Due to the virtual absence of EPR signals, intrinsic silicon constitutes a well-suited support for EPR studies.

Reactive sputtering of TiO_2_ is performed as previously reported by Graillot-Vuillecot et al.^200^ Deposition parameters are summarized in Table S1. X-ray diffraction indicates that the as-deposited TiO_2_ films are amorphous. However, after thermal treatment at 873 K either in high vacuum (*p* < 10^–6^ mbar) or in oxygen atmosphere (*p* = 650 mbar), the films undergo recrystallization, leading to the formation of the anatase crystallographic phase.

### Sample Characterization

Scanning electron microscopy (SEM) images are obtained using a ZEISS Ultra Plus field-emission scanning electron microscope operating at a voltage of 5 kV. Transmission electron microscopy (TEM) data are obtained using a JEOL JEM-F200 cold field-emission transmission electron microscope (Jeol Ltd., Tokyo, Japan) operating at 200 kV. Images are recorded using a TVIPS F216 2k by 2k CMOS camera (TVIPS GmbH, Gauting, Germany), and the samples are measured on lacey copper grids coated with carbon. Electron paramagnetic resonance (EPR) spectroscopy is performed with a Bruker EMXplus-10/12/P/L X-band spectrometer equipped with a waveguide Cryogen-Free System from Oxford Instruments. The spectra are recorded at 15 K with a field modulation frequency of 100 kHz, a modulation amplitude of 0.1 mT, a microwave excitation power of 1 mW, and a microwave frequency of ∼9.30 GHz. Spin quantification is carried out with Xenon software from Bruker.

## Results

Four different nanoparticle architectures (NPAs) were prepared to investigate how potential carbon impurities resulting from organic additives used for slurry formulation and interfaces formed upon sintering of nanoparticle ensembles determine the type and concentration of paramagnetic electron traps in random nanoparticle networks (Table 1). An anatase TiO_2_ nanoparticle powder prepared by metal organic chemical vapor synthesis served as a precursor material. The nanoparticles were purified by a suitable thermal annealing procedure to remove adsorbates such as organic species, yielding carbon-free oxide surfaces. The clean powder (NPA1) was converted into a nanoparticle network via drop casting. For this purpose, an aqueous nanoparticle slurry (0.30 mol·L^–1^ TiO_2_, 0.05 mol·L^–1^ Triton-X, and 0.30 mol·L^–1^ acetylacetone) was prepared. The slurry was then immobilized onto two different substrates: a bare silicon wafer (NPA3) or a silicon wafer covered by a dense, polycrystalline TiO_2_ film (NPA4). Finally, immobilized nanoparticle layers and, for comparison, an unsupported dried particle ensemble (NPA2) were sintered at 723 K in the oxygen atmosphere (for details, see Methods section). Some relevant properties of the four different NPAs are given in Table 1.

**Table 1:**
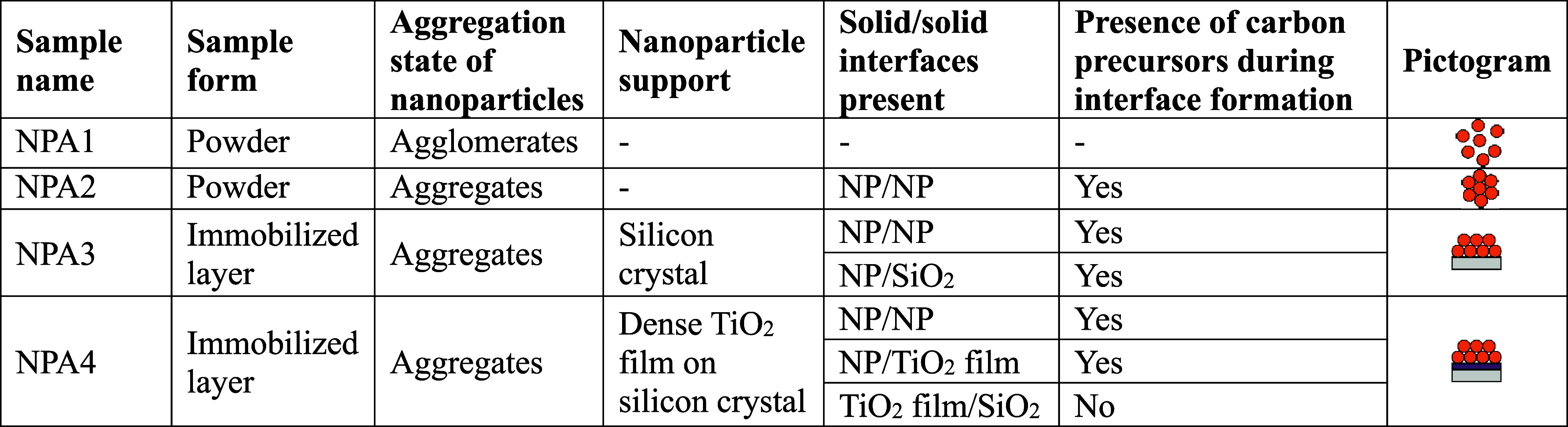
Properties of Different Nanoparticle Architectures (NPAs) Investigated in This Work

Additional characteristic features of the TiO_2_ nanoparticle-based configurations are summarized here with reference to earlier work.^10^ The basic structural component corresponds to the individual TiO_2_ anatase nanoparticles that were produced by metal–organic chemical vapor synthesis.^18^ In the absence of H_2_O and other solvent molecules, particle nucleation and growth leads to an average nanoparticle diameter, determined via transmission electron microscopy (TEM), and to a crystallite domain size, determined via X-ray diffraction, of 15 and 13 nm, respectively. After the multistep purification process in oxygen atmosphere and vacuum, the particles are agglomerated (NPA1), i.e., connected to each other only by weak particle–particle interactions (Figure 2a).

**Figure 2:**
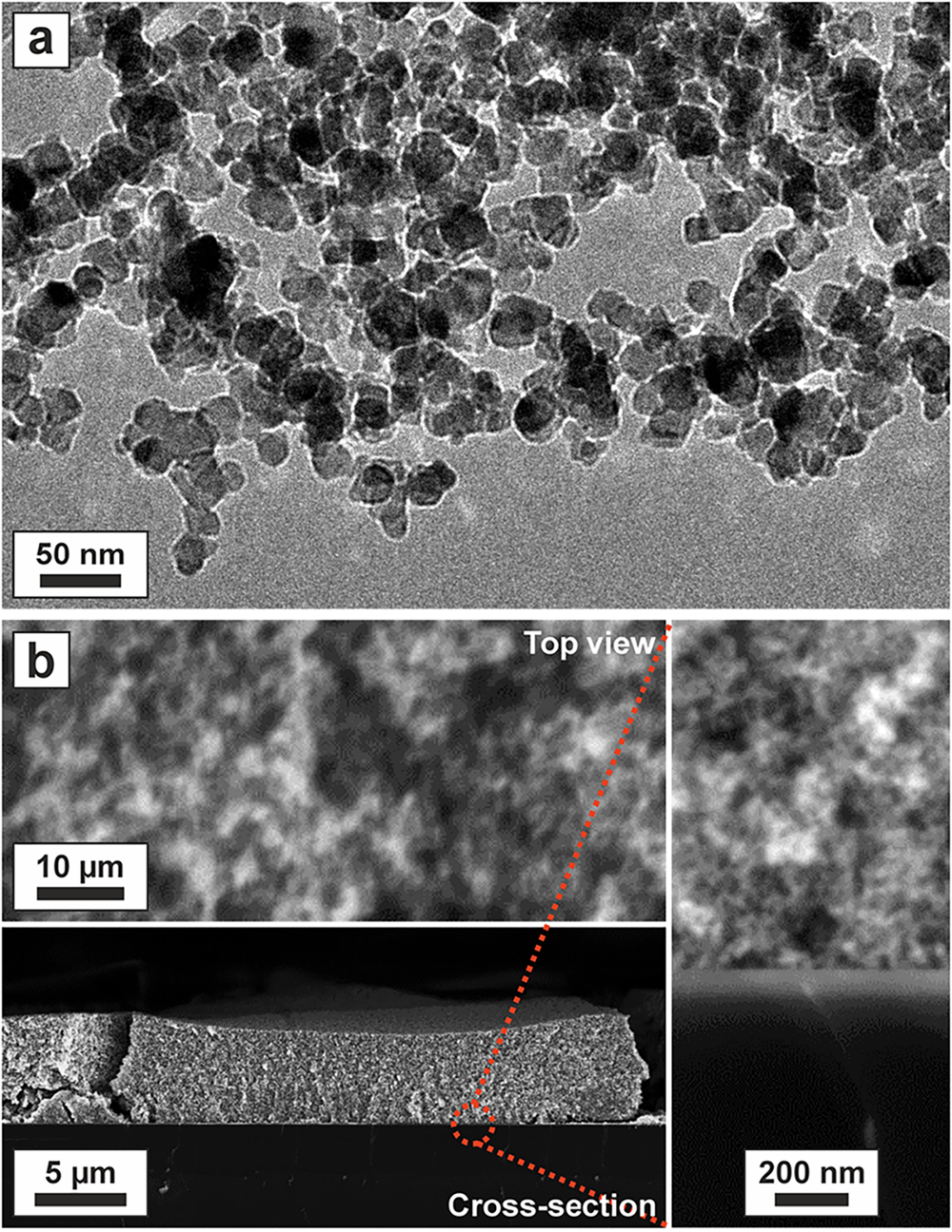
(a) TEM image of TiO_2_ nanoparticles (NPA1). (b) SEM top view and cross-sectional images of a composite structure formed by a cast TiO_2_ nanoparticle film on top of a TiO_2_ sputtered film (NPA4).

The formation of particle necks and solid–solid interfaces, which leads to rigid nanoparticle networks, can be achieved by immersing the dry nanoparticle powder into liquid water.^14,18^ Water present at the grain surfaces and interfaces reduces interparticle electrostatic repulsive forces.^19,20^ As a result, the particles can come into contact. Subsequent drying, dehydration, and sintering steps, which proceed upon thermal sample annealing, induce chemical bonding between the individual particles, enable neck formation and thus establish the connectivity of the nanoparticle network. Such a colloidal processing of preformed nanoparticle powders constitutes, therefore, a frequently used strategy for the fabrication of supported mesoporous layers (to be used as electrodes or photocatalysts).^1^ In this approach, an appropriate slurry formulation, which typically involves the use of organic additives, is a prerequisite for the preparation of nanoparticle layers featuring a homogeneous thickness and lacking macroscopic flaws (such as cracks).

In addition to the TiO_2_ nanoparticle powder obtained after purification treatment (NPA1, Figure 2a), we therefore investigated TiO_2_ nanoparticle networks (NPA2, NPA3, and NPA4) resulting from the thermal annealing of slurry-derived nanoparticle ensembles. The addition of organic additives (Triton-X and acetylacetone) to the aqueous dispersion was found to be essential to guarantee a sufficiently strong adhesion of the TiO_2_ nanoparticle network to the substrates (i.e., the bare or covered Si crystal). Such a slurry composition is routinely used for the preparation of mesoporous layers that are used for many applications, ranging from photo­(electro)­catalysis, sensing, to solar cells.^4,21,22^ Calcination of the dried nanoparticle ensembles at 723 K in an oxygen atmosphere is then performed to eliminate synthesis- and processing-related carbonaceous contaminants.

Figure 2b shows SEM images of a nanoparticle layer deposited on top of a sputtered compact TiO_2_ film with a thickness of approximately 100 nm (NPA4). On both substrates used in this work, i.e., pristine Si wafer (NPA3), and Si wafer coated with a thin TiO_2_ film (NPA4), drying of the cast nanoparticle dispersion yielded a uniform ca. 5 μm thick layer made of a network of interconnected TiO_2_ nanoparticles (Figure 2b). Furthermore, we determined comparable nanoparticle sizes for all three slurry-derived samples (NPA2, NPA3, and NPA4). Respective values are only slightly larger than for the nanoparticles of the precursor powder (NPA1).

Excess electrons have been introduced into the different NPAs upon oxygen removal from the TiO_2_ lattice at 973 K and under high vacuum. EPR spectroscopy was then used to characterize EPR-active electron traps.

As a dry powder (NPA1), which corresponds to the simplest material configuration, TiO_2_ nanoparticles are agglomerated and weakly interconnected. After vacuum annealing of NPA1 powder at 973 K, characteristic EPR signals of trapped electrons at titanium sites, i.e., Ti^3+^ ions, can be identified.^23–25^ Such defects correspond to polarons, and their signals feature *g*-factors below *g* = 2.0000 (signals above 3350 G in Figure 3a).

**Figure 3:**
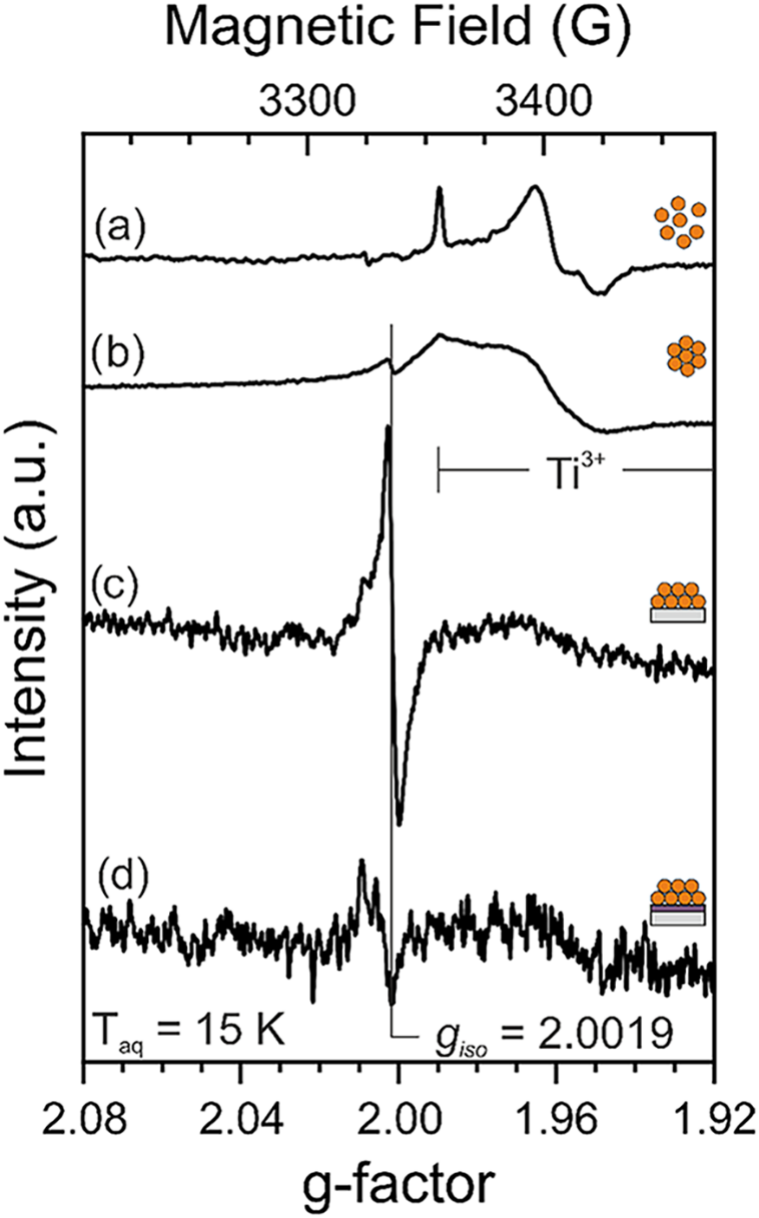
X-band CW-EPR spectra of vacuum-annealed (*T*_anneal_ = 973 K) (i.e., reduced) TiO_2_ nanoparticle architectures: (a) TiO_2_ nanoparticles (NPA1), (b) unsupported TiO_2_ nanoparticle networks (NPA2), and TiO_2_ nanoparticle networks on (c) a bare Si crystal (NPA3) or (d) 100 nm sputtered TiO_2_ film (NPA4). Spectra were recorded at *p* < 10^–5^ mbar. The signal intensity was referenced to the respective nanoparticle mass (mass-weighted intensity).

It is well documented that EPR spectra of oxygen-depleted TiO_2_ powders contain more than one single signal.^24^ Indeed, it has been shown that isolated Ti^3+^ sites at the surface or in the bulk regions of TiO_2_ nanoparticles, which give rise to characteristic *g*-tensors, constitute only a minor fraction of the reduced centers generated by thermal reduction.^13,26^ The largest fraction of centers contributes to a very broad and unstructured signal (at *g* = 1.93 and extending over more than 300 G). This signal has been attributed to a collection of surface and subsurface Ti^3+^ centers. Their heterogeneity was rationalized by differences in the local coordination of the ion. This contribution can be clearly seen in all the NPAs (Figure 3). More importantly, comparable concentrations of superoxide anions (Table S2) as detected after electron transfer to molecular oxygen (see below) point to a very similar degree of sample reduction for the thin film architectures NPA3 and NPA4 (a slightly higher reduction degree was observed for NPA1 and results from a higher surface area of these nonaggregated nanoparticles).

In addition to Ti^3+^ centers, we observe for all slurry-derived NPAs (i.e., NPA2–4) an isotropic signal at *g*_iso_ = 2.0019. A similar signal was recently reported for different types of TiO_2_ nanoparticle-based samples and was attributed to carbon impurity-based electron traps at the contact area between aggregated nanocrystals.^10^ The fact that thermal annealing in an oxidative atmosphere does not accomplish complete carbon impurity elimination is attributed to their location within the buried nanoparticle/nanoparticle (NP/NP) interface.^10^ Such an interpretation is in line with the presence of carbon sources during NP/NP interface formation in samples NPA2–4 (Table 1). Remarkably, however, the nanoparticle mass-weighted intensities of the signal at *g*_iso_ = 2.0019 significantly vary between NPA2, NPA3, and NPA4. Specifically, the intensities are comparable for NPA2 and NPA4. It is, however, much higher for NPA3. We now compare in more detail the corresponding mass-weighted intensities recorded for NPA3 and NPA4. Indeed, the only difference in the preparation and final geometry of NPAs 3 and 4 is the absence/presence of a barrier layer (i.e., the sputtered and dense TiO_2_ film) between the TiO_2_ nanoparticle network and the silicon substrate. If the centers contributing to the isotropic signal would result (for both architectures) exclusively from the TiO_2_ nanoparticle network, then we would expect the same signal intensity (at least after accounting for minor differences in the geometrical area of the layers). The intensity (as well as the mass-weighted intensity) of the signal is, however, a factor of ∼2 higher for NPA3 (Table 2). Remarkably, the (lower) mass-weighted signal intensity of NPA4 is comparable to the one of NPA2 (i.e., of the TiO_2_ nanoparticle aggregate powder, which can be considered as the pulverized TiO_2_ nanoparticle network of NPA2). Obviously, centers contributing to the isotropic signal measured for NPA4 (as well as NPA2) result exclusively from the TiO_2_ nanoparticle-based phase (i.e., the nanoparticle network or aggregates, respectively).

**Table 2:** Number of Carbon Impurity-Based Trapping Sites after Vacuum Annealing of the TiO_2_ Nanoparticle Architectures NPA3 and NPA4

	NPA3	NPA4
N_trapping sites_ · g^–1^	(1.8 ± 0.2)·10^16^	(1.0 ± 0.2)·10^16^
N_trapping sites_ · particle^–1^	0.07 ± 0.01	0.04 ± 0.01

The much higher mass-weighted intensity observed for NPA3 then serves as a first clear indication that for this type of nanoparticle architecture, more than one type of paramagnetic carbon impurity-based trapping site contributes to the signal at *g*_iso_ = 2.0019. At this point, we speculate that due to similar *g*_iso_ values, these different contributions must result from the same type of local defect (i.e., carbon impurity-based electron traps), which is located, however, at different interfaces within the nanoparticle architecture. Specifically, we point to the presence of two different carbon-exposed interfaces in NPA3, namely, TiO_2_/TiO_2_ and TiO_2_/silicon (or TiO_2_/SiO_2_) interfaces (Table 1). In line with such an interpretation, only TiO_2_/TiO_2_ interfaces are exposed (during interface formation) to carbon sources in NPA4. In these samples, the respective carbon sources are absent during the formation of the buried TiO_2_/SiO_2_ interface, provided that the Si crystal was properly cleaned prior to sputter deposition of the dense, polycrystalline TiO_2_ layer.

It is well-known that molecular oxygen can act as an acceptor for electrons from Ti^3+^ centers. Correspondingly, after oxygen adsorption and interfacial electron transfer, superoxide anions O_2_^–^ form, as clearly demonstrated by three-component EPR powder signals with resonance features at values *g* > 2.0000. The specific *g*-tensor components (*g*_*xx*_, *g*_*yy*_ and *g*_*zz*_) related to the orthorhombic O_2_^–^ signal are indicated in Figure 4 by lines. The resonances in the *g*_*zz*_ range, as indexed in Figure 4, contain information about the type and number of adsorption sites.^27^ The latter are surface cations that bind the O_2_^–^ ions in a symmetrical side-on configuration. The values for the measured *g*_*zz*_ resonances, which were determined to be in the range between 2.025 and 2.019, differ slightly between TiO_2_ nanoparticle agglomerates (NPA1) and slurry-derived nanoparticle aggregates (NPA2–4). This points to a modification of available adsorption sites upon colloidal processing.

**Figure 4:**
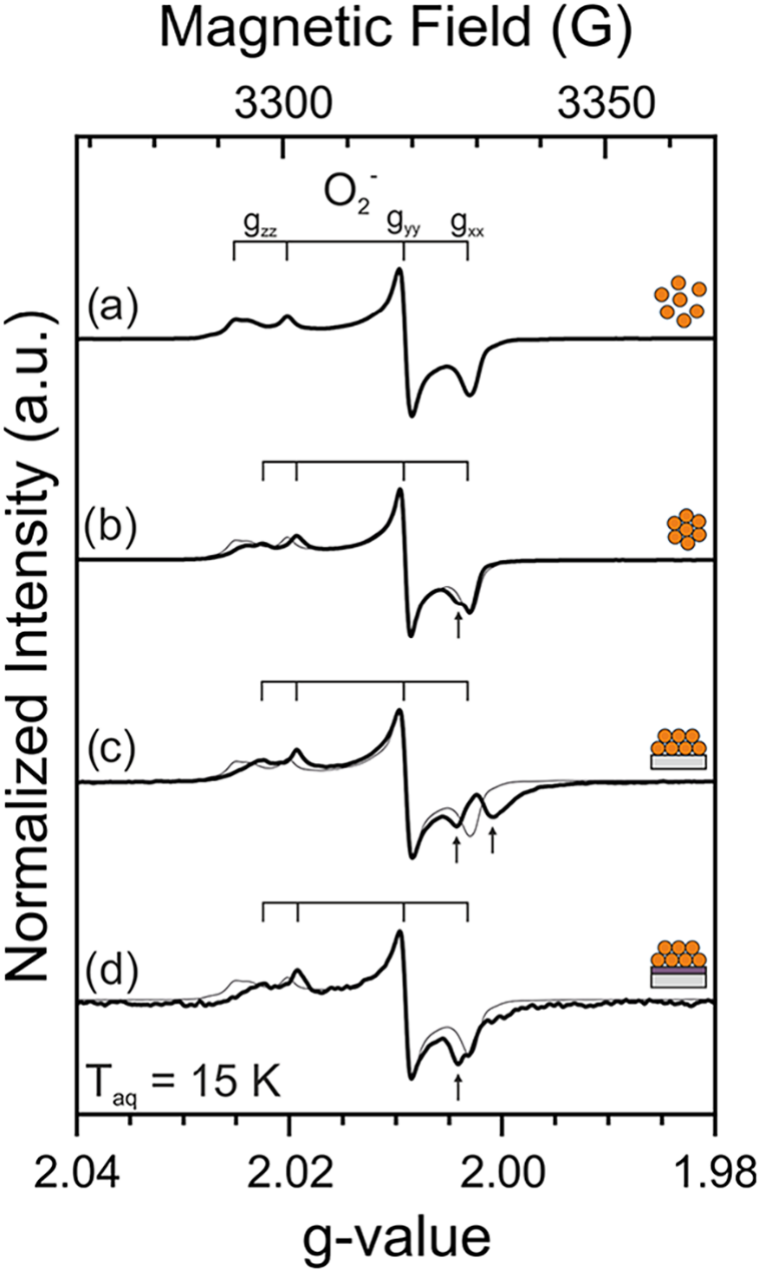
X-band CW-EPR spectra recorded after oxygen addition to vacuum-annealed (i.e., reduced) TiO_2_ nanoparticle architectures: (a) TiO_2_ nanoparticles (NPA1), (b) unsupported TiO_2_ nanoparticle networks (NPA2), and TiO_2_ nanoparticle networks that are either supported on (c) a bare Si crystal (NPA3) or (d) a 100 nm sputtered TiO_2_ film (NPA4). For the sake of comparison, the intensities of the spectra were normalized to equal peak-to-peak values of the *g*_*yy*_ component. Spectra (b–d) also contain the thin-lined envelope of (a). Following sample exposure to oxygen at room temperature, the system was pumped down to a base pressure of *p* < 10^–5^ mbar.

The range of *g*_*zz*_ values between 2.025 and 2.019 indicates a distribution of different adsorption sites for the side-on coordinated surface radicals present on the TiO_2_ nanoparticle surfaces exposed.^27,28^

For TiO_2_ nanoparticles (NPA1, Figure 4a), the EPR signal envelope points to superoxide anions as the only type of paramagnetic species present after oxygen addition. This indicates that all paramagnetic electron traps (specifically, the Ti^3+^ centers) are involved in the interfacial electron transfer to molecular oxygen. The result, however, does not exclude the presence of (EPR-silent) diamagnetic electron centers after oxygen addition. Furthermore, other (though diamagnetic) products may result from the interfacial electron transfer.^29^

In line with our previous study,^10^ we observe that oxygen addition to the vacuum-annealed (i.e., lattice oxygen-deficient and reduced) unsupported TiO_2_ nanoparticle network (NPA2) shifts the *g*-factor of the isotropic signal (attributed to carbon impurity-based electron traps at NP/NP interfaces) from *g*_iso_ = 2.0019 to 2.0050 (Figure 4b). This shift is, however, more pronounced than the oxidation-induced shift (from *g*_iso_ = 2.0019 to 2.0028) observed in the previous study.^10^ We attribute this observation to the involvement of organic additives, where the corresponding residues may slightly alter the local geometrical and electronic structure of the NP/NP interfaces. In any case, this shift can be explained by the partial reoxidation of the reduced oxide upon oxygen admission.^10^ NPA4, i.e., TiO_2_ nanoparticle network supported on the sputtered layer of dense and polycrystalline TiO_2_ behaves very similar in this respect (NPA4, Figure 4d). Additional isotropic signals present for slurry-derived samples (NPA2–4) are indicated by arrows in Figure 4.

The most interesting behavior related to the effect of oxygen exposure, however, is observed for the TiO_2_ nanoparticle network supported on the bare silicon crystal (NPA3, Figure 4c). Indeed, we identify for this sample (in addition to the superoxide anion signal) two isotropic signals at *g*_iso_ = 2.0019 and 2.0050.

This result is a second strong indication for the contribution of more than one type of paramagnetic carbon impurity-based trapping site to the signal at *g*_iso_ = 2.0019 in the case of reduced NPA3 (Figure 3c). The reoxidation of the oxide, which takes place upon the addition of molecular oxygen to oxygen-deficient TiO_2_ at room temperature and which is irreversible with regard to sample evacuation, obviously alters the local electronic structure of (sub)­surface defects, resulting in a change of the resonance position. Correspondingly, for carbon impurity-based electron traps at TiO_2_/TiO_2_ interfaces (both NP/NP and NP/TiO_2_ film, Table 1), a shift of the *g*-value from *g*_iso_ = 2.0019 to 2.0050 (as estimated from the displacement of the (first derivative) EPR signal’s minimum) is observed (in line with NPA2 and NPA4, Figure 4b,d). To fully rationalize the observed strong impact of such a sample modification on both *g*-values (and possibly the relaxation behavior) of the investigated spin center, one has to consider its location at particle/particle contact areas. In such sample regions, local strain and electrostatic perturbations significantly impact the properties of defects, and we expect them to experience significant changes upon sample reoxidation.^30^ These local effects are, however, extremely difficult to quantify even when using model interfaces.^8^

Such a reoxidation upon oxygen exposure will, however, not take place in the SiO_2_ phase (expected to be present at the NP/SiO_2_ interface of NPA3) as SiO_2_ is an irreducible oxide. The *g*-value of traps associated with an SiO_2_ phase is therefore expected to be invariant upon oxygen exposure. We therefore attribute the isotropic signal (observed upon oxygen exposure of NPA2–4) at *g*_iso_ = 2.0050 to carbon impurity-based electron traps, i.e., *carbonecks*, at TiO_2_/TiO_2_ interfaces. On the other hand, we attribute the isotropic signal (observed upon oxygen exposure of NPA3) at *g*_iso_ = 2.0019 to carbon impurity-based electron traps at TiO_2_/SiO_2_ (i.e., NP/SiO_2_) interfaces.

In Figure 5 the EPR spectra of silicon-supported cast nanoparticle films (NPA3) recorded at different microwave excitation levels are depicted. In addition, the two isotropic signals (*g*_iso_ = 2.0019 and 2.0050) and their relative intensity are indicated by arrows, while the superoxide anion (O_2_^–^) specific *g*-tensor components (*g*_*xx*_, *g*_*yy*_ and *g*_*zz*_) are indicated by lines. The characteristic orthorhombic EPR powder signals related to O_2_^–^ in Figures 4 and 5 are based on the *g-*factor components with values listed in Table 3.

**Table 3:** *g*-Value Components Related to the Orthorhombic EPR Powder Signals of O_2_^–^ Ions Isolated on TiO_2_ Nanoparticle Powders, TiO_2_ Nanoparticle Networks (after Nanoparticle Aggregation), and Cast TiO_2_ Nanoparticle Films (Figures 4 and 5)

	*g*-tensor values		
	*g_zz_*	*g_yy_*	*g_xx_*
NPA1	2.025(0); 2.020(2)	2.009(1)	2.003(1)
NPA2	2.022(5); 2.019(3)	2.009(1)	2.003(1)
NPA3	2.022(5); 2.019(3)	2.009(1)	2.003(1)
NPA4	2.022(3); 2.019(3)	2.009(1)	2.003(1)

**Figure 5:**
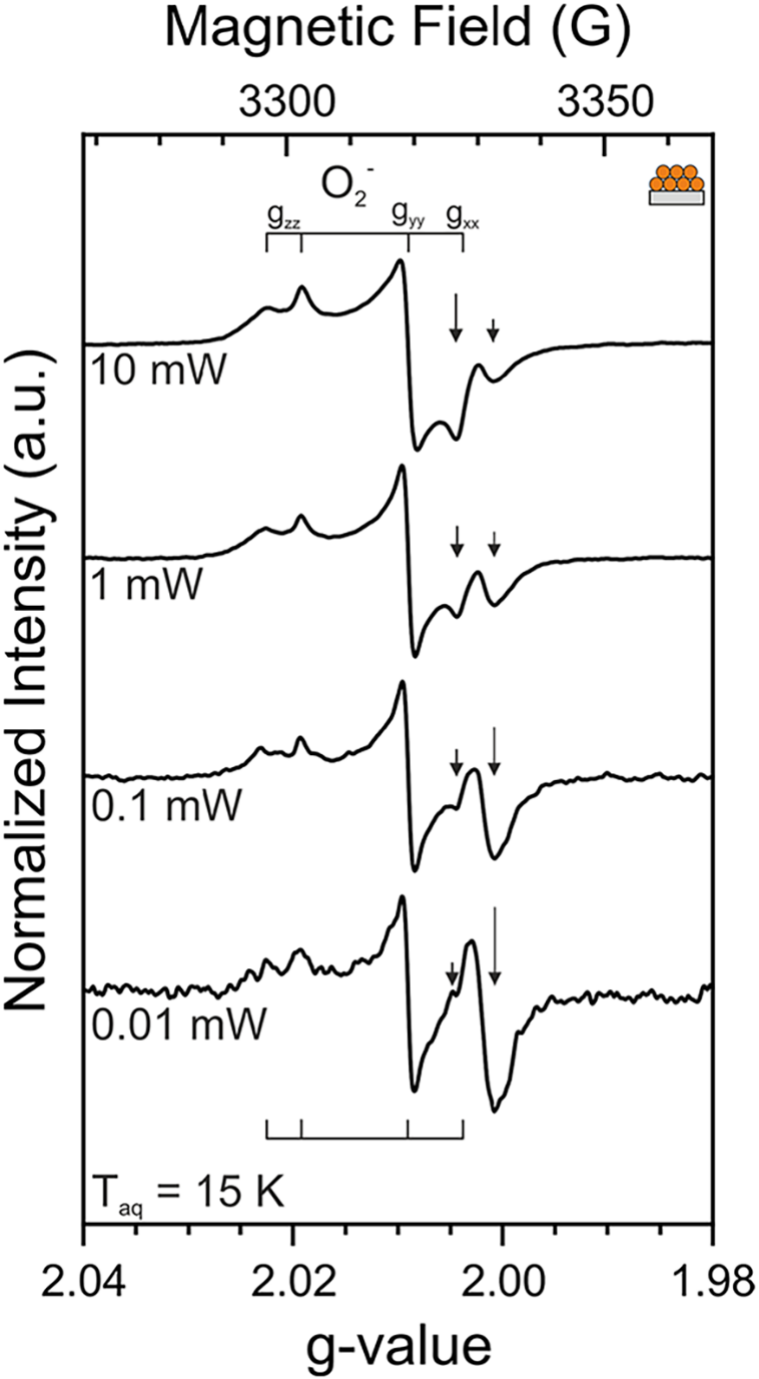
Normalized X-band CW-EPR spectra recorded after oxygen addition to a vacuum-annealed (i.e., reduced) TiO_2_ nanoparticle network supported as a 5 μm thick layer on a bare Si crystal (NPA3). Following sample exposure to oxygen at room temperature, the system was pumped down to a base pressure of *p* < 10^–5^ mbar.

As the microwave power is reduced, the normalized intensity of the signal at *g*_iso_ = 2.0050 decreases. Conversely, the signal intensity at *g*_iso_ = 2.0019 increases. Thus, the microwave power saturation behavior of the two isotropic signals at *g*_iso_ = 2.0019 and 2.0050 is very different, which supports the previous conclusion that the corresponding carbon impurity-based electron traps are embedded into electronically very different environments.

To summarize, in addition to paramagnetic superoxide anions, we identified carbon impurity-based electron traps at TiO_2_/TiO_2_ and TiO_2_/SiO_2_ interfaces with *g*-values of 2.0050 and 2.0019, respectively. The presence of these traps depends on both the nanoparticle-based architecture and the materials processing parameters used to prepare the nanostructured films. We consider both aspects as highly relevant for the processing and application of nanoparticle-based materials. Nanoparticle necking is a process that occurs naturally during nanoparticle powder processing. During this process, even ambient carbon atoms can dissolve in water and become entrapped at the interfaces between nanoparticles.^10^ The incorporation of carbon atoms into the anionic sublattice of TiO_2_, i.e., the formation of *carboneck* centers, occurs during powder processing at elevated temperature. This process is explained by the fact that attachment and fusion of the particles are also part of the dynamic processes that occur during particle ensemble purification and oxidative carbon removal. As a result, traces of carbon become trapped in the contact areas between the particles. Upon particle coalescence, these convert from surface species into bulk species. Even after extensive chemical and thermal post-treatment steps, *carbonecks* inside TiO_2_ nanomaterials remain at a concentration level of a few ppm.^10^

This phenomenon is particularly relevant for functional materials since they are often fabricated as supported films and, to improve the homogeneity and adhesion of the film to the substrate, organic additives are used.^9–11^ Previous studies have addressed the presence of carbon in TiO_2_-based nanostructured photocatalysts. EPR signals with *g*-values between 2.003 and 2.004 have been reported and associated with substitutional carbon or intercalated carbon centers within the nanostructured TiO_2_ host lattice.^12,13,30,31^ The present study adds further important insights into the often-overlooked impact of synthesis- and processing-related impurities on the properties of nanoparticle-based materials.

## Conclusions

Paramagnetic defects are strongly influenced by the TiO_2_ nanoparticle architecture and the processing conditions. The casting process, which involves carbon sources, results in the entrapment of carbon impurities at the TiO_2_/TiO_2_ and TiO_2_/SiO_2_ interfaces. Thermal annealing in an oxidative atmosphere does not eliminate these impurities, indicating that they are located at buried interfaces. Upon oxygen exposure, the electronic environment of the defects at the TiO_2_/TiO_2_ interface changes, causing a shift in the *g*-value. Conversely, EPR signals attributed to electron traps at the TiO_2_/SiO_2_ interface remain invariant upon oxygen exposure. The distinct electronic environment of carbon-related defects leads to unique saturation behaviors. Importantly, depositing a TiO_2_ sputtered film on the silicon substrate before casting acts as a protective layer, preventing the formation of trapping centers at the TiO_2_/SiO_2_ interface. Our work provides a detailed EPR study of the defects that can form during the preparation of cast TiO_2_ nanoparticle films. As such, it should be relevant for many different research groups working with nanostructured TiO_2_ films, which have a wide range of applications.

## Supplementary Material



## Notes

EPR spectra of as-received and thermally processed Si crystals as well as the synthesis parameters for TiO_2_ deposition via reactive sputtering (PDF)
