# Supplementary material for: Carbon Impurity Entrapping and Charge Localization within TiO2 Nanoparticle Films
Source: J Phys Chem C Nanomater Interfaces. 2025 Oct 29;129(45):20180–7. doi: 10.1021/acs.jpcc.5c04882 (PMC12621236; doi:10.1021/acs.jpcc.5c04882)
Supplement: Supplementary file 1 [file jp5c04882_si_001.pdf]

Supporting Information

Carbon Impurity Entrapping and Charge Localization within TiO_2 Nanoparticle Films

Guillem Vives Ollé[a], Gilles R. Bourret[a], Thomas Berger[a] and Oliver Diwald[a]

[a] Department of Chemistry and Physics of Materials, Paris-Lodron University Salzburg, Jakob-Haringer-Straße 2a, A-5020 Salzburg, Austria

E-mail: gilles.bourret@plus.ac.at, thomas.berger@plus.ac.at

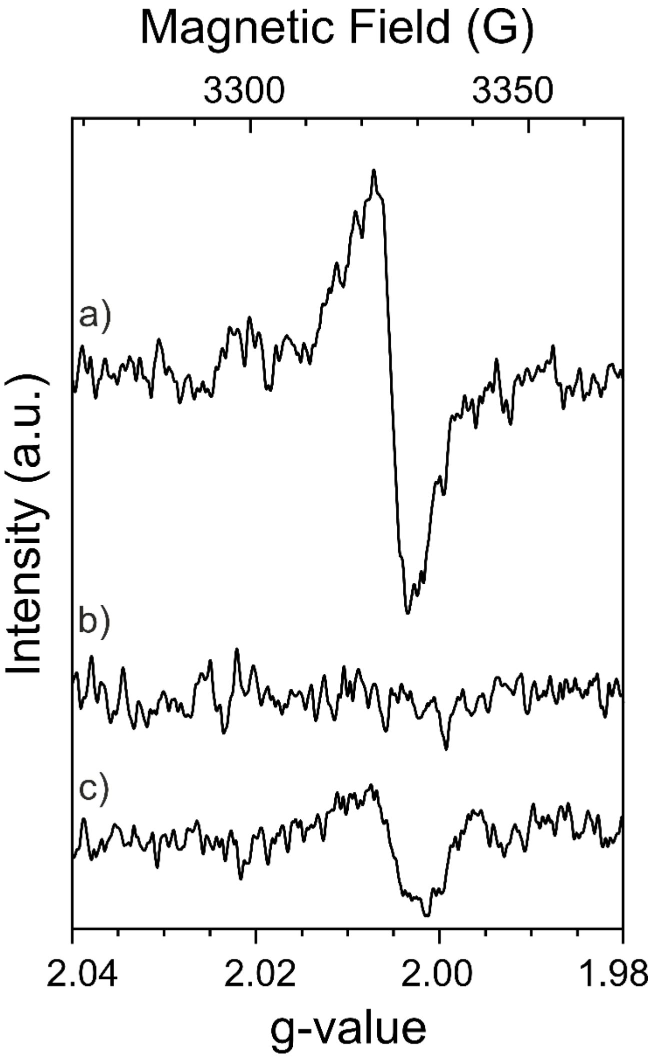

Figure S1: EPR spectra of (a) an as-received Si crystal, (b) a Si crystal after vacuum annealing at 973 K, and (c) after subsequent annealing at 873 K in oxygen atmosphere.

Table S1: Synthesis parameters for TiO_2 deposition via reactive sputtering.

**Sputtering Gas**	Ar	**Ar Gas Flow**	20 sccm
**Secondary Gas**	O_2	**O_2 Gas Flow**	3 sccm
**Power**	50 W	**Deposition time**	1 hour
**Chamber pressure**	$1.1 \cdot 10^{-3}$ mbar	**Target information**	Titanium ⌀ 152.4 mm (Bühler)

Table S2: Number of superoxide anions (O_2^-) after vacuum annealing and subsequent oxygen exposure of the TiO_2 nanoparticle architectures NPA3 and NPA4

	**NPA3**	**NPA4**
$\mathbf{N_{trapping\ sites} \cdot g^{-1}}$	$(2.8 \pm 1.2) \cdot 10^{17}$	$(3.8 \pm 1.5) \cdot 10^{17}$
$\mathbf{N_{trapping\ sites} \cdot particle^{-1}}$	1.15 ± 0.34	1.44 ± 0.55
